# Global burden of viral skin diseases from 1990 to 2021: a systematic analysis for the global burden of disease study 2021

**DOI:** 10.3389/fpubh.2025.1464372

**Published:** 2025-02-19

**Authors:** Deng Li, Ming Chen, Wei Li, Xuewen Xu, Qingfeng Li

**Affiliations:** ^1^Department of Plastic and Burns Surgery, West China Hospital, Sichuan University, Chengdu, China; ^2^Department of Plastic & Reconstructive Surgery, Shanghai Ninth People's Hospital Affiliated to Shanghai Jiaotong University School of Medicine, Shanghai, China

**Keywords:** viral skin diseases, disability-adjusted life-years, global burden of disease, incidence, prevalence, trend, age-period-cohort

## Abstract

**Background:**

The scarcity of knowledge regarding the epidemiology and temporal patterns of viral skin diseases worldwide poses significant challenges to their control and management.

**Methods:**

We analyzed the global incidence, prevalence, and age-standardized rates (ASR) of disability-adjusted life years (DALYs) for viral skin diseases in 2021. To examine temporal trends from 1990 to 2021, we employed the EPAC model, assessing changes by country, gender, age, Socio-demographic Index (SDI), and GBD regions. Additionally, we utilized the age-period-cohort (APC) model and the Bayesian age-period-cohort (BAPC) model to forecast the burden of viral skin diseases for the next 25 years.

**Results:**

In 2021, the global burden of viral skin diseases was estimated at 84.7 million incident cases, with a prevalence of over 130 million cases and 4.2 million DALYs. Males experienced a slightly higher ASR burden than females. The highest burden was observed among individuals aged 10 to 19, with significant geographical variations in cases and ASR, particularly in high SDI regions. Unexpected rises in incidence were noted in East Asia and Sub-Saharan Africa in the detected period. Despite modest declines in ASPR and ASDR, the global ASIR displayed a significant upward trend.

**Conclusion:**

Our study provides detailed data on the global impact of viral skin diseases from 1990 to 2021, highlighting the need for continuous surveillance and tailored interventions to manage and reduce the effects of these diseases. Targeted public health measures are essential to address and mitigate the global health burden of viral skin diseases.

## 1 Introduction

Viral skin diseases are among the most common dermatological affections worldwide and involve people of different regions, ethnicities, and socioeconomic levels (1, 2). Smallpox represented a significant chapter in the history of global public health, demonstrating the immense burden of viral diseases, which had severe systemic effects along with characteristic skin manifestations (3). In the modern era, the spread of mpox has once again underscored the vulnerabilities in global health and highlighted the persistent threat posed by these infections (4). Among these, human herpesvirus, viral warts and molluscum contagiosum are counted among the top 50 most prevalent diseases globally (5, 6). “Infections caused by herpes simplex viruses (HSV), including HSV-1 and HSV-2, represent a significant global health burden”. In 2016, approximately 66.6% of the global population under 50 had infections with HSV-1, and 13.2% of the population aged 15–49 had infections with HSV-2 (6).These diseases pose significant challenges to public health due to their high morbidity rates, DALYs, and economic costs, particularly affecting pediatric populations. The epidemiology of viral skin diseases is closely associated with demographic shifts (7, 8), socioeconomic development (9), ethnicity (10) and vaccination campaigns such as the measles (11), mumps (12), and rubella vaccine (13, 14).

Despite advancements in early detection and treatment modalities, profound changes in global population structures and socioeconomic factors over the past three decades have significantly impacted the incidence and burden of viral skin diseases (15). Specifically, regions with lower incidence rates or limited resources often need more comprehensive research on the sources and trends of these diseases, hampering the implementation of effective control measures (16).

The Global Burden of Disease (GBD) collaboration has established itself as a leading non-governmental organization in shaping the global landscape of health metrics. It assesses the attributable burden of various risk factors across all countries (17, 18). However, a significant gap remains in our understanding of the burden posed by viral skin diseases. This study seeks to bridge that gap by estimating the global incidence, prevalence, and DALYs of viral skin diseases in 2021, while also analyzing trends from 1990 to 2021. This comprehensive analysis will lay a strong foundation for informed policymaking and tailored prevention efforts, ultimately supporting the development of effective public health strategies and interventions.

## 2 Methods

### 2.1 Data source

This research quantified the impact of diseases or disorders in terms of DALYs, which measure the total years lost due to illness, disability, or early death within a population (19). The GBD 2021 data offered annual insights into viral skin diseases across global, regional, and national levels from 1990 to 2021. This extensive dataset included information on new cases, prevalence, DALYs, and their respective age-standardized incidence rates (ASIR), prevalence rates (ASPR), and DALY rates (ASDR), organized by gender and age and accessed through the Global Health Data Exchange (GHDx) query tool (20, 21).

Viral skin diseases were classified using the International Classification of Diseases (ICD-10: B09) (22). To estimate Years of Life Lost (YLLs) and Years Lived with Disability (YLDs) from prevalence data, specific disability weights were applied, considering the severity of the disease and associated comorbidities. The disease burden, including incidence and YLDs, was quantified using DisMod-MR 2.1, a Bayesian meta-regression tool. ST-GPR utilized spatial and temporal data to refine risk factor exposure and mortality rate estimates. Data sources, which included 95% uncertainty intervals (UIs), comprised cancer registries, published studies, surveillance data, census data, and other relevant sources customized to specific locations, genders, and age groups (23). DALYs were calculated by combining YLLs and YLDs for each cause (24).

### 2.2 Statistical analyses

To determine temporal trends of burden for viral skin diseases, estimated annual percentage change (EAPC) of ASIR, ASDR, and ASPR between 1990 to 2021 were calculated globally. The calculation has been widely established in the literature. The annual per cent change for ASR was estimated with a regression line model, with the dependent variable as the natural logarithm of the rates [ln(ASR)] and the independent variable as the calendar year. EAPC for ASR was calculated as 100 × [exp(*β*)–1], with its corresponding 95% confidence interval (CI) (25, 26).

For forecasting these metrics from 2021 to 2046, we ran the age-period-cohort (APC) model along with the Bayesian age-period-cohort (BAPC) model, using an integrated nested Laplace approximation (INLA) framework to estimate marginal posterior distributions. This method addresses common issues of mixing and convergence encountered in traditional Markov Chain Monte Carlo (MCMC) sampling techniques and for its computational efficiency and its ability to quickly handle large, complex datasets, which is crucial for our global study. The analysis utilized the BAPC and INLA packages within R version 4.3.1 (27).

### 2.3 Geographical analysis and data presentation

The GBD 2021 methodology to estimate causes of death and diseases remains broadly consistent with that used in GBD 2019 but is updated with new data inputs and methodologic improvements. This is to ensure that the produced results by GBD 2021 are better in quality than those produced by previous iterations of the GBD. Reporting for the study follows the Guidelines for Accurate and Transparent Health Estimates Reporting (GATHER).

GBD 2021 evaluated the incidence, prevalence, mortality, YLDs, YLLs, and DALYs for different age groups and sexes across 204 countries and territories, grouped into 21 regions and seven super-regions. Subnational analyses were performed for specific countries, with the GBD location hierarchy expanded to encompass all WHO member states by including new countries and territories. The analytical framework produced annual estimates from 1990 to 2021, organized around diseases and injuries in a hierarchy with a structure covering the categories from the top level through the detailed ones. Metadata and data are made available for public access through the Global Health Data Exchange (GHDx).

The SDI is a composite measure of lag-distributed income per capita, average years of education, and fertility rate for women under 25. Annualized rates of change were calculated as the difference in the natural logarithm of values at the start and end of a period divided by the number of years in that period. This method was used to investigate the association of SDI with the annualized rate of change in age-standardized DALY rates for all causes. Their removal eliminates the possibility that they will overpower the trends of other causes.

## 3 Results

### 3.1 Worldwide incidence, prevalence and DALYs of viral skin diseases in 2021

In 2021, the global impact of viral skin diseases was distinguished, with a total incidence of 84.73 million cases (95% UI: 82.13 to 87.59 million), reflecting a notable prevalence across sexes and all age strata. This translated to a prevalent caseload of 136.81 million (95% UI: 133.16 to 140.72 million) cases, contributing to approximately 4.20 million DALYs (95% UI: 2.67 to 6.26 million). These collective findings underscore the substantial global impact of viral skin diseases, affecting a wide demographic range.

A gender-stratified analysis revealed that males exhibited a slightly higher age-standardized rate (ASR) for prevalence and DALYs than females, whereas females had a marginally lower incidence of ASR. Specifically, in 2021, the incidence among females was 41.31 million cases (95% UI: 40.03 to 42.68 million), corresponding to an ASR of 1120.03 (95% UI: 1083.85 to 1158.68). The prevalent caseload among females stood at 64.47 million (95% UI: 62.79 to 66.18 million), with an ASR of 1699.6 (95% UI: 1653.31 to 1748.62). The estimated DALYs for females were 1.97 million (95% UI: 1.26 to 2.94 million), translating to an ASR of 52.09 (95% UI: 33.24 to 77.75).

Conversely, males reported an incidence of 43.42 million cases (95% UI: 42.08 to 44.84 million), yielding an ASR of 1133.24 (95% UI: 1097.43 to 1171.22). The prevalent caseload among males was 72.34 million (95% UI: 70.24 to 74.55 million), with an ASR of 1861.96 (95% UI: 1808.71 to 1919.04). The estimated DALYs for males were 2.23 million (95% UI: 1.41 to 3.33 million), corresponding to an ASR of 57.41 (95% UI: 36.4 to 85.77).

**Figure 1:**
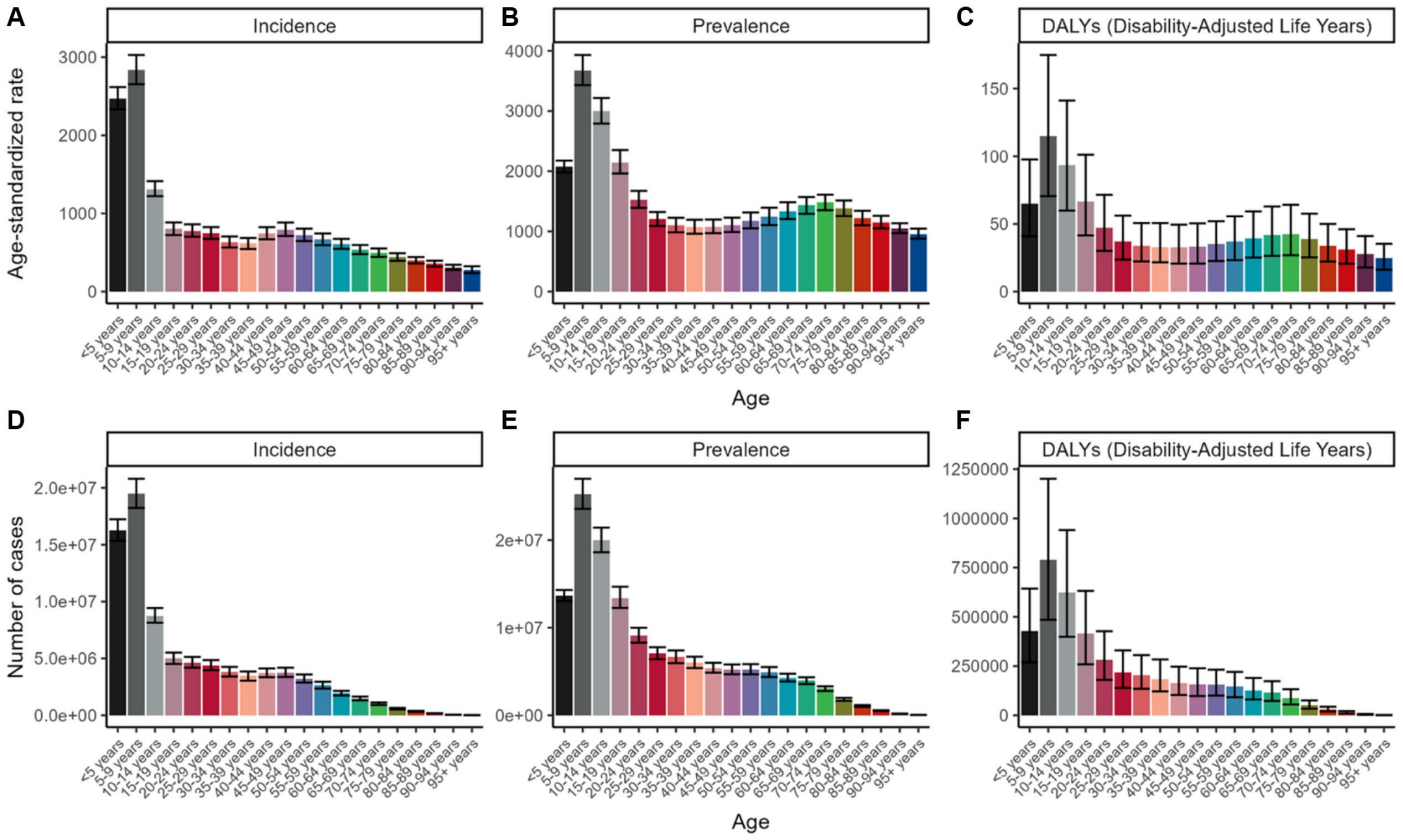
Age-standardized rate per 100,000 population, for incidence, prevalence, and DALYs of viral skin diseases globally in 2021, delineated by age group. **(A)** ASR of incidence; **(B)** ASR of prevalence; **(C)** ASR of DALYs; **(D)** Number of incidence cases; **(E)** Number of prevalence cases; **(F)** Number of DALYs cases.

An age-specific analysis further revealed that the highest incidence rates were observed in children aged 10 to 19, with a subsequent significant decline in older age groups. A modest rise in incidence was noted within the 40–54 age group, while both prevalence and DALY rates slightly increased around 65 (Figure 1).

### 3.2 Geographic disparities in the incidence, prevalence, and burden of viral skin diseases in 2021

In 2021, notable differences in the incidence, prevalence, and burden of viral skin diseases were evident across various regions and countries. These disparities followed a distinct pattern linked to the SDI. Regions with higher SDI levels exhibited more significant numbers of viral skin disease cases and higher ASRs for incidence, prevalence, and DALYs. In contrast, these figures progressively declined in areas with lower SDI levels. Interestingly, the lowest values were recorded in low-middle SDI regions rather than the weakest areas. Among all countries, High-income North America emerged as a significant outlier, reporting the highest ASRs for incidence, prevalence, and DALYs, significantly surpassing other nations.

**Figure 2:**
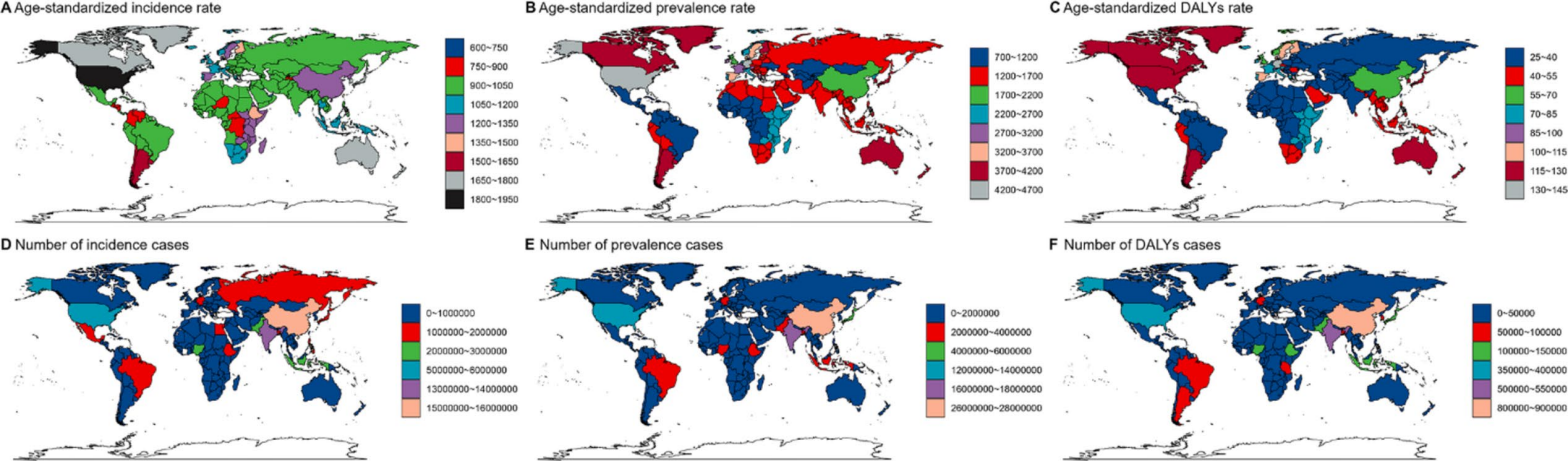
Global distribution of age-standardized rates (ASR) for viral skin diseases in 2021, showing incidence **(A)**, prevalence **(B)**, and DALYs **(C)**, alongside the total number of incidence cases **(D)**, prevalence cases **(E)**, and DALYs cases **(F)**.

Figure 2 illustrates the differences in ASRs for incidence, prevalence, and DALYs of viral skin diseases across 204 countries and territories in 2021, revealing significant variations. The ASR of incidence ranged widely, from the lowest value of 888.98 (95% UI: 857.43 to 929.67) per 100,000 individuals in Central Sub-Saharan Africa to the highest of 1804.09 (95% UI: 1763.02 to 1841.39) in High-income North America. Similarly, the prevalence of ASR exhibited a broad spectrum, varying from 920.15 per 100,000 in Central Sub-Saharan Africa (95% UI: 885.85 to 957.05) to 4207.32 in High-income North America (95% UI: 4139.1 to 4274.95). The ASRs for DALYs also showed marked fluctuations, ranging from 28.29 in Central Sub-Saharan Africa (95% UI: 18.09 to 41.54) to 128.64 in High-income North America (95% UI: 82.15 to 192.64) per 100,000 individuals, reflecting a fourfold difference between these countries.

**Figure 3:**
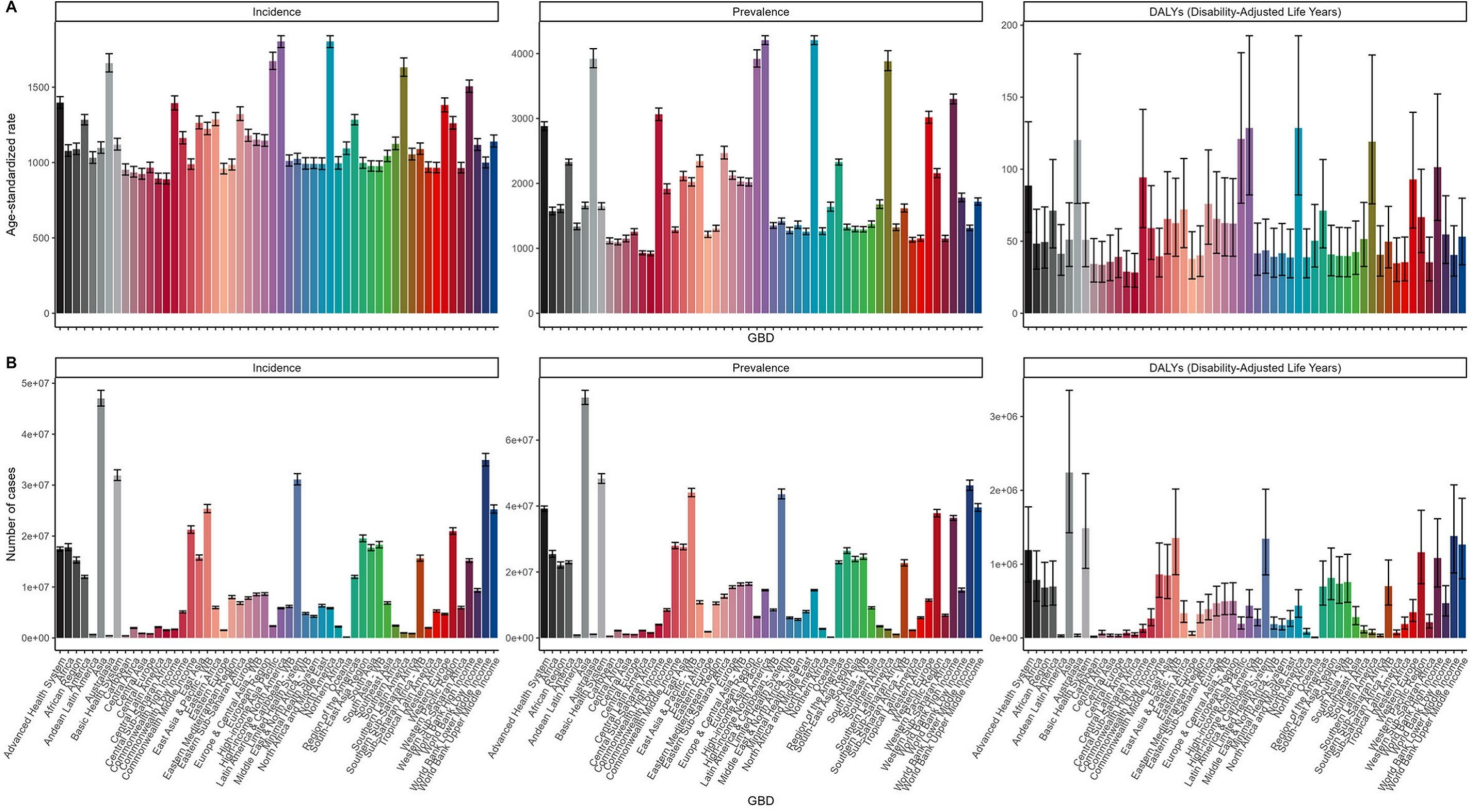
Geographic Disparities in ASIR, ASPR, and ASDR of Viral Skin Diseases in 2021. **(A)** Age-standardized incidence rate (ASIR), age-standardized prevalence rate (ASPR), and age-standardized DALY rate (ASDR) of viral skin diseases across different regions. **(B)** Total number of incidence, prevalence, and DALY cases across different regions.

Figure 3 highlights the disparities in viral skin disease burden across various regions. In Figure 3A, the ASR of incidence peaks in high-SDI regions, exceeding 1,500 per 100,000 individuals, while prevalence rates are highest in North America and parts of Europe, surpassing 4,000 per 100,000. DALYs are most pronounced in parts of South America and Africa, reaching over 150 per 100,000. Figure 3B shows that the highest number of incidence and prevalence cases are observed in densely populated countries like China and India, with over 50 million and 60 million cases, respectively. DALYs also peak in low-SDI regions, exceeding 3 million cases.

The total number of incidence and prevalence cases exhibited considerable variation, peaking in densely populated countries like China and India. These countries contributed substantially to the global disease burden due to their vast populations. Specifically, the number of incidence cases spanned from under 10,000 in smaller nations to over 10 million in China. Analogously, total prevalence cases ranged from less than 20,000 in certain countries to over 30 million in China. Notably, DALYs also displayed significant variability, with higher values observed in regions with elevated incidence and prevalence rates, such as parts of Africa and South America, indicating a considerable disease burden in these areas.

### 3.3 Temporal trends in the incidence, prevalence, and burden of viral skin diseases from 1990 to 2021

**Figure 4:**
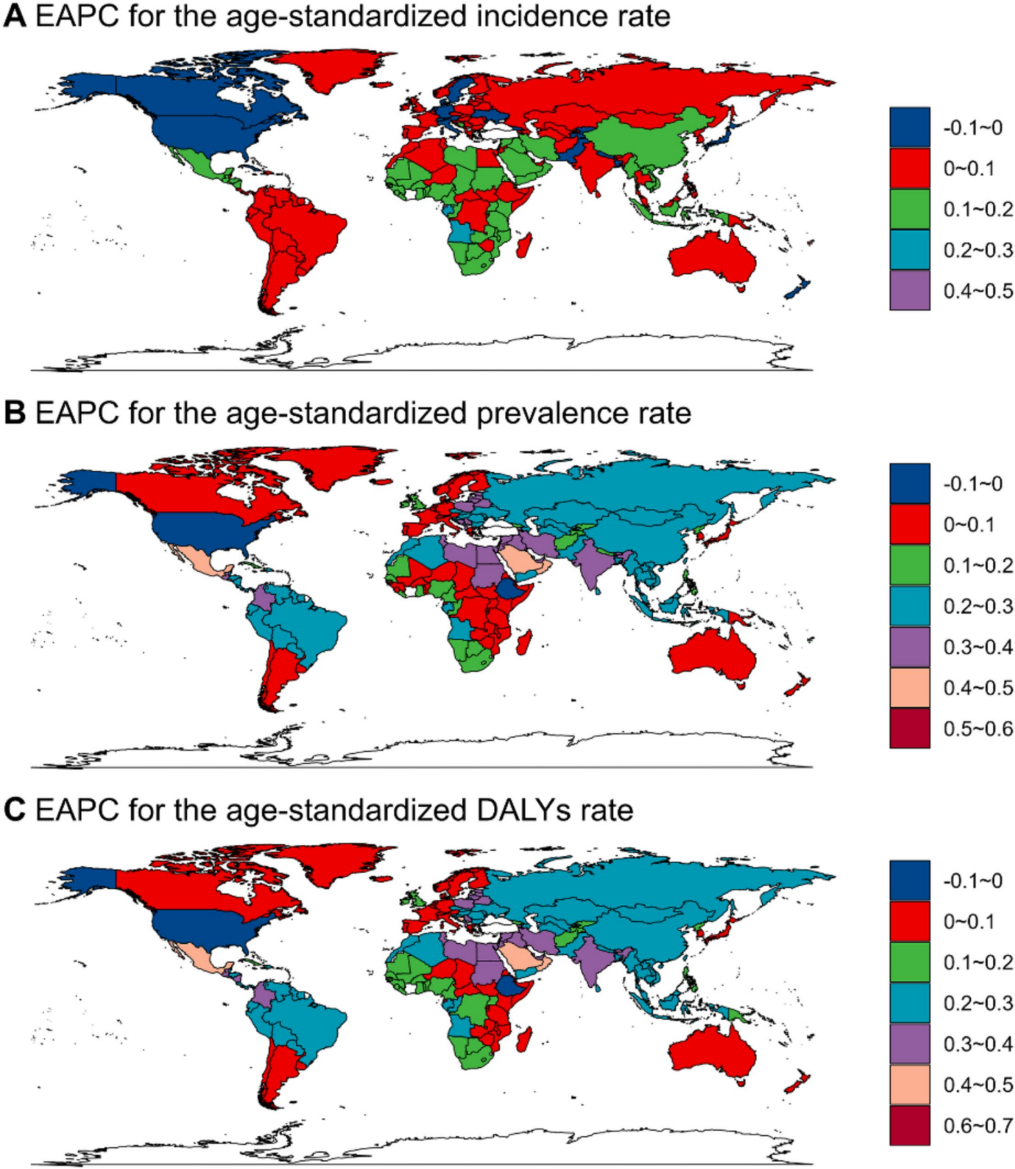
EAPCs of ASR from 1990 to 2021 for viral skin diseases, illustrating the global distribution of ASR for incidence **(A)**, prevalence **(B)**, and DALYs **(C)**. White-colored countries indicate missing or unavailable data for the specified EAPC range.

From 1990 to 2021, while the global number of viral skin disease cases rose, the ASRs for both incidence and prevalence exhibited a slight decline (Table 1; Figure 4), particularly in regions with high SDI scores, including parts of North America and Europe. Conversely, regions with low SDI scores, such as those in Africa and Central Asia, experienced decreases in incidence and prevalence rates (Figures 4A,B), highlighting a growing disparity in disease burden between areas with different socioeconomic statuses. White-colored countries in Figures 4A–C indicate missing or unavailable data for the respective EAPC ranges.

**Table 1:** Incidence, prevalence and DALYs of global viral skin diseases in 2021 and the temporal trends from 1990 to 2021.

	2021						1990–2021		
	Incidence		Prevalence		DALYs		ASIR	ASPR	ASDR
Characteristics	Numbers (95% UI)	ASR	Numbers (95% UI)	ASR	Numbers (95% UI)	ASR	(95% CI)	(95% CI)	(95% CI)
		No. ×10–5 (95%UI)		No. ×10–5 (95%UI)		No. ×10–5 (95%UI)	EAPC_CI	EAPC_CI	EAPC_CI
Global									
Both	84,730,445 (82132966–87,586,946)	1126.25 (1090.92–1165.91)	136,807,660 (133155771–140,724,829)	1781.31 (1733.08–1835.16)	4,199,075 (2671027–6,261,658)	54.77 (34.83–81.77)	−0.03 (−0.03--0.03)	−0.05 (−0.06−−0.04)	-0.04 (−0.05−−0.03)
Female	41,305,808 (40031577–42,681,949)	1120.03 (1083.85–1158.68)	64,466,306 (62789423–66,180,793)	1699.6 (1653.31–1748.62)	1,970,117 (1257418–2,935,310)	52.09 (33.24–77.75)	-0.03 (−0.04--0.03)	−0.08 (−0.09−−0.07)	-0.07 (−0.08--0.06)
Male	43,424,637 (42076603–44,835,509)	1133.24 (1097.43–1171.22)	72,341,353 (70236759–74,547,273)	1861.96 (1808.71–1919.04)	2,228,958 (1413609–3,326,779)	57.41 (36.4–85.77)	−0.02 (−0.03−−0.02)	-0.02 (−0.03−−0.01)	-0.01 (−0.02–0)
SDI region									
High SDI	13,958,350 (13625969–14,278,411)	1559.42 (1519.01–1600.76)	32,877,827 (32167170–33,493,867)	3370.24 (3292.88–3445.9)	996,743 (635616–1,486,466)	103.55 (65.83–155.35)	−0.04 (−0.05--0.03)	0 (0–0.01)	0 (0–0.01)
High-middle SDI	12,579,252 (12206986–12,996,027)	1168.95 (1130.66–1213.35)	22,211,100 (21563037–22,827,104)	1948.17 (1888.68–2010.59)	681,405 (430420–1,015,796)	60.27 (38.1–90.17)	0.12 (0.1–0.13)	0.25 (0.24–0.27)	0.26 (0.24–0.27)
Middle SDI	24,227,949 (23478237–25,057,677)	1077.53 (1041.99–1117.21)	34,725,955 (33697502–35,824,898)	1511.06 (1465.27–1561.02)	1,069,737 (681657–1,605,411)	46.67 (29.76–70.23)	0.02 (0.02–0.03)	0.05 (0.03–0.07)	0.06 (0.04–0.08)
Low-middle SDI	19,684,858 (19003959–20,441,361)	994.5 (960.29–1033.08)	26,048,144 (25183304–26,948,829)	1305.33 (1264.58–1348.41)	803,930 (512328–1,209,797)	40.2 (25.63–60.46)	0.07 (0.06–0.07)	0.26 (0.25–0.28)	0.28 (0.27–0.3)
Low SDI	14,220,707 (13717927–14,783,306)	1056.98 (1022.11–1094.94)	20,860,281 (20062095–21,760,573)	1569.97 (1515.51–1632.25)	644,663 (408389–968,396)	48.24 (30.49–71.89)	0.07 (0.06–0.08)	0.07 (0.06–0.07)	0.09 (0.08–0.1)
High-income Asia Pacific	2,333,117 (2260593–2,397,928)	1673.13 (1617.75–1732.16)	6,329,872 (6147187–6,507,787)	3919.74 (3792.17–4058.11)	192,003 (121088–285,210)	120.99 (76.38–180.86)	0 (0–0.01)	0.06 (0.06–0.06)	0.06 (0.06–0.06)
Central Asia	898,499 (864240–935,021)	924.23 (889.35–961.73)	1,098,272 (1050980–1,148,593)	1149.74 (1100.11–1203.37)	34,099 (21604–51,769)	35.69 (22.64–54.3)	0.01 (0–0.02)	0.23 (0.21–0.25)	0.24 (0.22–0.26)
East Asia	15,786,099 (15287681–16,288,739)	1263.58 (1223.23–1309.35)	27,560,321 (26737238–28,432,587)	2111.41 (2043.05–2185.59)	847,239 (534069–1,266,212)	65.45 (41.2–98.28)	0.14 (0.13–0.15)	0.2 (0.2–0.21)	0.22 (0.21–0.22)
South Asia	17,733,793 (17131863–18,337,692)	977.12 (942.44–1011.85)	23,853,034 (23142333–24,723,816)	1293.77 (1255.45–1339.22)	734,784 (469242–1,100,324)	39.81 (25.42–59.61)	0.01 (0.01–0.01)	0.32 (0.3–0.34)	0.34 (0.32–0.36)
Southeast Asia	6,857,154 (6627561–7,092,261)	1043.06 (1006.3–1081.54)	9,124,716 (8814500–9,451,556)	1373.27 (1326.38–1422.49)	282,110 (179514–425,217)	42.48 (26.99–64.04)	0.1 (0.1–0.11)	0.21 (0.2–0.22)	0.22 (0.22–0.23)
Australasia	435,979 (421569–451,214)	1659.73 (1600.96–1722.11)	1,122,409 (1083277–1,162,094)	3919.86 (3782.33–4075.05)	34,081 (21680–50,645)	120.24 (76.25–180.04)	0.01 (0–0.01)	0.06 (0.06–0.06)	0.06 (0.06–0.07)
Caribbean	419,725 (404767–436,335)	952.64 (916.99–991.3)	498,985 (480981–519,326)	1113.3 (1070.64–1161.8)	15,323 (9707–23,212)	34.26 (21.69–51.85)	0.01 (0.01–0.02)	0.17 (0.17–0.18)	0.17 (0.17–0.18)
Central Europe	803,737 (779868–828,843)	966.09 (933.15–1002.26)	1,015,071 (984704–1,044,842)	1256.61 (1211.92–1303.32)	31,308 (19902–46,691)	39.11 (24.76–58.68)	0.04 (0.03–0.05)	0.3 (0.29–0.31)	0.31 (0.3–0.32)
Eastern Europe	1,518,105 (1469740–1,565,989)	959.63 (924.55–994.8)	1,888,176 (1821374–1,956,196)	1213.94 (1167.63–1262.91)	58,205 (37096–87,102)	37.72 (23.87–56.62)	0.02 (0.01–0.02)	0.27 (0.26–0.29)	0.28 (0.26–0.3)
Western Europe	4,691,562 (4554850–4,829,527)	1381.66 (1335.63–1428.66)	11,470,688 (11164024–11,782,662)	3018.69 (2926.21–3110.74)	348,749 (222266–520,170)	92.91 (59.08–139.5)	−0.09 (−0.11--0.07)	0 (0–0.01)	0 (0–0.01)
Andean Latin America	674,010 (649988–699,709)	1031.97 (994.68–1072.17)	878,469 (845910–912,590)	1336.78 (1287.79–1387.65)	27,133 (17314–40,457)	41.28 (26.33–61.57)	0.06 (0.06–0.07)	0.21 (0.2–0.22)	0.22 (0.21–0.23)
Central Latin America	2,127,980 (2053404–2,208,178)	893.99 (860.94–929.5)	2,255,820 (2182116–2,326,864)	932.26 (900.68–962.28)	69,683 (44535–105,023)	28.84 (18.41–43.44)	0.09 (0.08–0.1)	0.34 (0.32–0.37)	0.35 (0.33–0.38)
Southern Latin America	994,867 (960447–1,031,847)	1631.77 (1572.31–1694.27)	2,530,751 (2438758–2,630,051)	3881.72 (3737.39–4044.52)	77,301 (49307–115,366)	119.15 (75.96–179.26)	0.02 (0.01–0.03)	0.06 (0.05–0.06)	0.06 (0.05–0.06)
Tropical Latin America	1,977,279 (1914757–2,047,314)	966.53 (934.94–1004.41)	2,343,616 (2276198–2,418,753)	1129.33 (1095.51–1168.88)	71,775 (45724–108,005)	34.72 (22.06–52.36)	0.08 (0.07–0.08)	0.24 (0.24–0.25)	0.25 (0.25–0.26)
North Africa and Middle East	6,318,659 (6084992–6,579,009)	990.48 (953.83–1031.4)	7,964,266 (7664850–8,312,833)	1257.88 (1211.75–1310.03)	245,621 (156369–370,648)	38.72 (24.64–58.39)	0.1 (0.09–0.11)	0.31 (0.3–0.32)	0.32 (0.31–0.33)
High-income North America	5,841,677 (5717471–5,961,023)	1804.09 (1763.02–1841.39)	14,493,630 (14282534–14,692,194)	4207.32 (4139.1–4274.95)	437,450 (280230–653,773)	128.64 (82.15–192.64)	−0.05 (−0.06--0.03)	−0.02 (−0.04–0)	−0.03 (−0.04--0.01)
Oceania	171,134 (164489–178,479)	1093.79 (1053.54–1136.85)	245,270 (234653–257,046)	1638.42 (1571.78–1712.28)	7,577 (4830–11,374)	50.32 (32.14–75.54)	0.01 (0.01–0.02)	0.08 (0.07–0.08)	0.09 (0.08–0.09)
Central Sub-Saharan Africa	1,526,167 (1464488–1,603,824)	888.98 (857.43–929.67)	1,540,667 (1476950–1,608,685)	920.15 (885.85–957.05)	47,669 (30390–70,721)	28.29 (18.09–41.54)	0.13 (0.1–0.16)	0.12 (0.1–0.15)	0.15 (0.12–0.19)
Eastern Sub-Saharan Africa	6,821,083 (6581581–7,095,349)	1321.56 (1279.08–1369.83)	12,609,612 (12090939–13,248,124)	2468.63 (2376.91–2572.02)	390,230 (246387–590,910)	75.9 (47.9–113.37)	0.1 (0.09–0.1)	0.02 (0.02–0.02)	0.05 (0.04–0.05)
Southern Sub-Saharan Africa	872,535 (841634–906,098)	1054.41 (1017.04–1094.91)	1,095,832 (1053830–1,140,728)	1323.01 (1274.34–1374.76)	33,730 (21385–50,429)	40.63 (25.79–60.75)	0.09 (0.08–0.09)	0.1 (0.1–0.11)	0.1 (0.1–0.11)
Western Sub-Saharan Africa	5,927,284 (5704770–6,194,795)	963.84 (931.16–1001.18)	6,888,182 (6577181–7,202,885)	1151.74 (1107.43–1200.51)	213,005 (134799–318,832)	35.41 (22.49–52.87)	0.15 (0.14–0.16)	0.12 (0.11–0.13)	0.15 (0.13–0.16)

No., number; DALYs, disability-adjusted-life-years; ASIR, age-standardized incidence rate; ASPR, age-standardized prevalence rate; ASDR, age-standardized DALY rate; UI, uncertainty intervals; CI, confidence interval; SDI, socio-demographic index; EAPC, estimated annual percentage change.

The ASR for DALYs saw the most notable increase in lower SDI regions, especially in parts of Africa and South America. In contrast, regions with high and middle SDI scores, such as North America and Europe, saw significant declines (Figure 4C). These disparities underscore the pronounced regional differences. For example, Western Africa recorded the sharpest rise in ASR for incidence, with an EAPC of 0.15 (95% CI: 0.14 to 0.16), whereas the smallest decline was noted in the United States-0.08 (95% CI: −0.09 to-0.07). The ASR for prevalence showed substantial variation, from a drop of-0.11 in the United States (95% CI: −0.14 to-0.09) to an increase of 0.34 in Central Latin America (95% CI: 0.32 to 0.37). Globally, the ASR for DALYs showed a slight decrease, with the most significant reduction observed in the United States (EAPC: -0.07, 95% CI: −0.08 to-0.06). In contrast, Equatorial Guinea had the most significant increase in DALYs (EAPC: 0.05, 95% CI: 0.04 to 0.06).

Figure 4 highlights the observed trends, with ASIR increasing in North America and certain parts of Europe while decreasing in Africa and Central Asia. Meanwhile, ASPR rose in Sub-Saharan Africa, some Asian regions, and South America but fell in North America, parts of Europe, and Australia.

### 3.4 Forecasting the future burden of viral skin diseases: insights from APC and BAPC models

**Figure 5:**
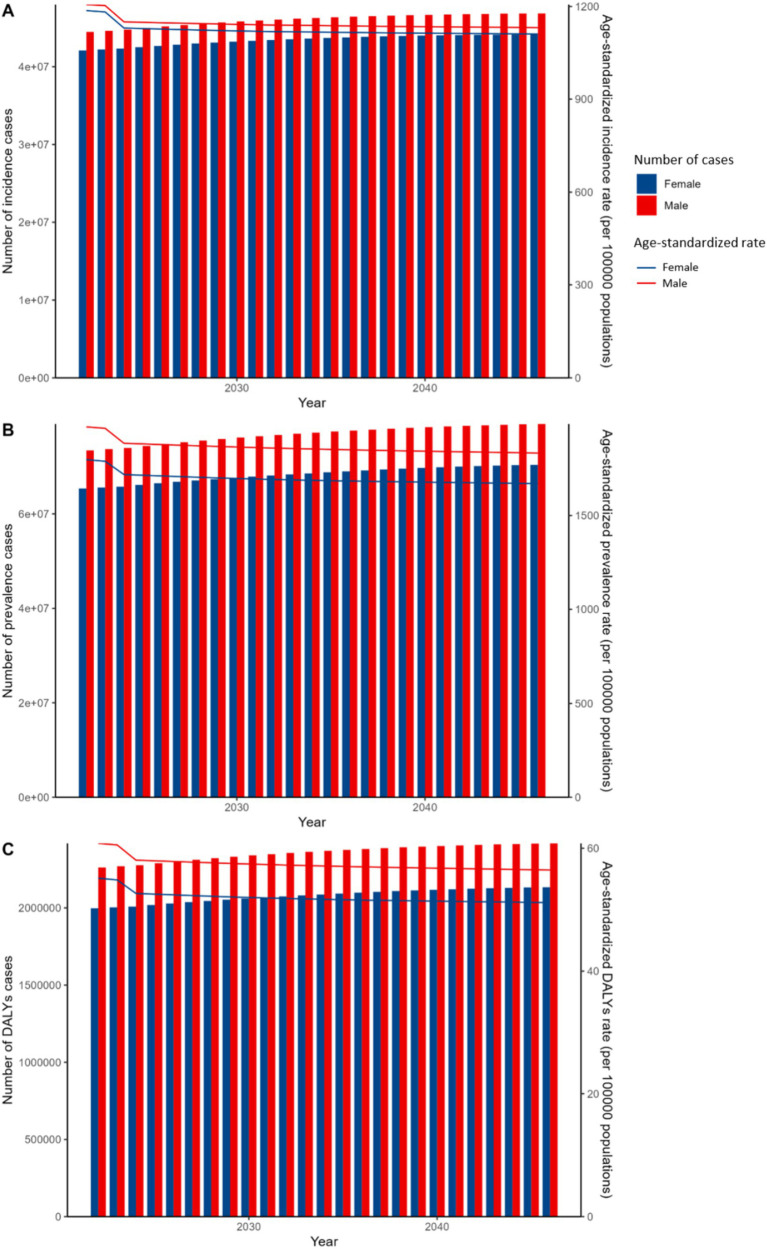
Projected APC global trends model of incidence, prevalence, and DALYs for viral skin diseases by sex, 2020–2050. **(A)** Incidence cases and ASIR. **(B)** Prevalence cases and ASR. **(C)** DALYs cases and ASDR. Bars show cases (left y-axis); lines show rates (right y-axis). Red: male, Blue: female.

The projected results from the APC model, which forecasts future trends in the ASIR, ASPR, and ASDR, stratified by gender, are summarized in Figure 5. These results indicate a projected rise in all indicators over the coming decades. However, the related ASRs are thought to remain relatively stable. The BAPC model confirms these findings, ensuring the stability of the projections. Both charts indicate notable gender differences, with health indicators higher in males compared to otherwise. Additionally, the trends remain consistent, showing a gradual increase in incidence rates, prevalence rates, and DALYs over the years, with males experiencing higher numbers of cases and rates than females.

**Figure 6:**
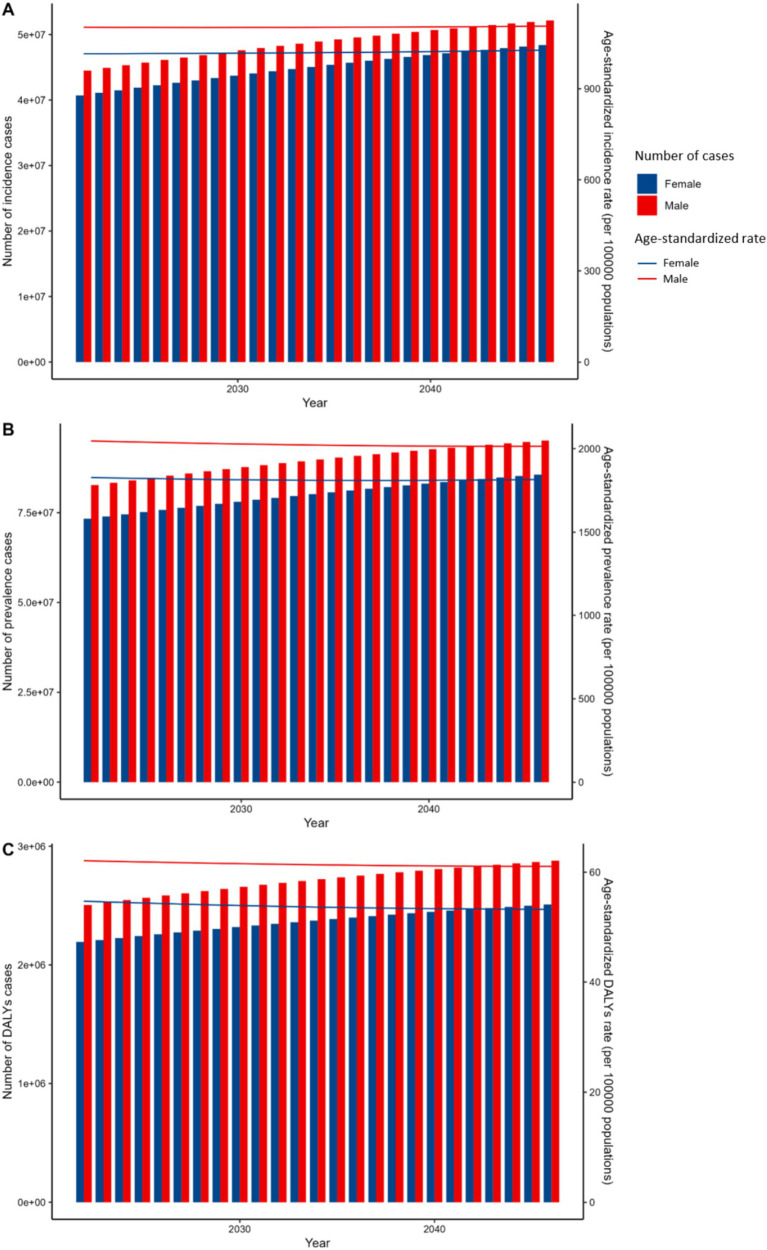
Projected BAPC global trends model of incidence, prevalence, and DALYs for viral skin diseases by sex, 2020–2050. **(A)** Incidence cases and ASIR. **(B)** Prevalence cases and ASR. **(C)** DALYs cases and ASDR. Bars show cases (left y-axis); lines show rates (right y-axis). Red: male, Blue: female.

Specifically, from 2021 to 2046, in the BAPC model, the ASIR for females is anticipated to remain broadly stable, hovering around 1,015 per 100,000 population. Similarly, males are expected to exhibit a comparable trend in incidence rates. Nevertheless, despite these seemingly stable rates, the number of incidence cases among females is predicted to increase from approximately 35.6 million in 2021 to 42 million by 2046. A comparable increase is anticipated for males. As shown in Figure 6A, the ASIR for males is projected to rise to approximately 1,100 per 100,000 population by 2030, while for females, it is expected to increase to around 1,000 per 100,000 population during the same period.

Based on the BAPC model shown in Figure 6B, the ASPR for females is expected to gradually decrease from 1828 per 100,000 in 2021 to around 1814 per 100,000 by 2030, and further to 1816 per 100,000 by 2046, indicating a relatively stable trend with a slight decline. A similar downward trend is observed for males, with ASPR decreasing from 2047 per 100,000 in 2021 to 2029 per 100,000 by 2030, and further to 2015 per 100,000 by 2046.

Regarding DALYs, the rates for females are forecasted to decrease slightly, from 54.7 per 100,000 in 2021 to 53.5 per 100,000 by 2046, with males displaying a comparable downward trend. Despite this modest reduction in DALYs, cases among females are anticipated to increase from approximately 2.5 million in 2021 to 3 million by 2046. Males are expected to follow a similar pattern. As depicted in Figure 6C, the ASDR for males is projected to rise to approximately 65 per 100,000 population by 2030, while for females, it is anticipated to increase to around 60 per 100,000 population by 2030.

## 4 Discussion

This study offers an in-depth examination of global, regional, and national trends in viral skin disease incidence, prevalence, and DALYs from 1990 to 2021. By breaking down the data by age and gender, we gained a thorough understanding of the burden and progression of these diseases over time. Through trend analyses, we sought to uncover patterns and insights to guide future research and inform public health strategies.

Monitoring the burden of viral skin diseases through DALYs provides a crucial resource for governments, donor agencies, international organizations, and civil society groups. This metric helps to pinpoint and prioritize emerging areas of concern, guiding targeted interventions and resource allocation (2, 28, 29). Our study underscores the substantial burden of these diseases among young children, with molluscum contagiosum particularly prevalent in this age group. Poor hygiene practices and contact with contaminated items, such as toys, clothing, towels, bedding, and swimming pools, exacerbate the risk of transmission in children, particularly in disadvantaged regions (15). As individuals age, the overall burden of viral skin diseases tends to decline, likely attributed to the development of immunity (30). However, we observed a slight increase in prevalence, incidence, and ASDR among middle-aged adults. This pattern may be explained by factors like immunosenescence or heightened exposure to risk factors during this life stage (24). The slight increase in ASIR, ASPR, and ASDR observed among middle-aged adults may be due to a combination of occupational environmental factors, increased social stress leading to systemic inflammation, and lifestyle behaviors prevalent in this age group. Middle-aged individuals are often exposed to various occupational hazards and may experience higher levels of stress due to professional and personal responsibilities (31). Chronic stress has been associated with immune system alterations, potentially increasing susceptibility to infections (32). Additionally, higher rates of sexual activity may contribute to an elevated risk of acquiring skin viral diseases (33). However, direct factors explaining this trend were not identified in our study, and these interpretations remain speculative. Further research is needed to elucidate the underlying causes of the observed increase in disease rates among middle-aged populations.”

Though often self-limiting, the necessity of active treatment for viral skin diseases remains a subject of debate. This debate stems from the diverse treatment options and the need for definitive evidence for the most productive approach. Active treatment is generally recommended for complications or cosmetic concerns (34). While retrospective studies indicate that aggressive treatment does not significantly enhance resolution rates, measures to prevent disease transmission among all patients remain crucial (35). In 2021, there was a global decline in the burden of viral skin diseases. This decrease can be attributed to the extensive adoption of preventive strategies, such as self-monitoring, public awareness campaigns, and various treatment modalities. These efforts have substantially reduced the worldwide impact of viral skin diseases (31).

The GBD study highlights significant regional differences in the impact of viral skin diseases, with socioeconomically disadvantaged regions bearing a greater burden. This emphasizes the need for customized public health strategies to mitigate these disparities and effectively decrease the worldwide prevalence of these diseases.

However, regions like Central Sub-Saharan Africa, Western Sub-Saharan Africa, and Central Latin America are experiencing a growing burden of viral skin diseases. These diseases are more commonly found in rural and densely populated areas. Countries with lower economic wealth often have lower vaccination coverage, heightening the risk of viral infections (36). Limited clean water, poor hygiene, and restricted medical care significantly contribute to transmission. In densely populated urban areas, factors like overcrowding, limited space, and poor living conditions facilitate direct transmission. Close contact and high density exacerbate infection spread. Regions with lower economic resources also face reduced vaccination coverage and weak public health infrastructure, increasing outbreak risk. The combination of poor sanitation, crowded conditions, and low immunization rates makes these areas vulnerable to viral skin diseases.

Between 1990 and 2021, the ASIR, ASPR, and ASDR for viral skin diseases demonstrated a declining trend in both sexes. However, males consistently faced a higher prevalence and burden of these diseases than females, with the gender gap not only persisting but also expanding over time. This notable gender disparity in health outcomes underscores the necessity for tailored, gender-specific policies and interventions to effectively prevent and manage viral skin diseases (37).

The implementation of these measures holds promise in not just reducing the disease burden but also fostering greater health equity. Notably, preventive initiatives, including public education and novel treatment modalities, have demonstrated significant effectiveness in high-prevalence and high-incidence regions like the United States, Ethiopia, and Nepal, contributing significantly to the reduction in ASDR.

The transmission of infectious diseases, including viral skin infections, is being increasingly influenced by climate change, potentially affecting the prevalence of certain viral infections, like tick-borne encephalitis and mosquito-borne disease in previously unaffected high-latitude regions (38, 39). Interestingly, the burden of viral skin diseases appears to rise with higher SDI levels, likely attributed to better healthcare facilities and an increased number of dermatologists capable of diagnosing viral skin infections. In contrast, in low-SDI countries, a significant proportion of viral skin infections likely remain undiagnosed, thereby contributing to a lower reported burden. A study conducted in 30 households in southwestern Ethiopia revealed that upon examination, 67% of unreported skin diseases were deemed treatable (40), highlighting the need for improved diagnostic capabilities in these regions.

The EAPC serves as a comprehensive indicator of the trends in age-standardized rates across specific time intervals. Globally, while the EAPC for the ASIR of viral skin diseases exhibited a decline, there was a noteworthy surge in typically low-incidence regions such as the Netherlands. This rise may be attributed to climate change, which is altering disease transmission patterns and prevalence in higher-latitude countries (41). Central and Western Sub-Saharan Africa exhibited the highest EAPC for age-standardized DALY rates in the detected period, while Western Europe, as well as high-income North America, experienced significant reductions. These findings highlight the necessity for targeted prevention and screening programs to reduce healthcare costs and disease incidence.

Policy revisions present significant prospects for health development in the 21st century. The future disease burden will hinge on advancements in risk reduction and improved access to effective interventions.

The APC and BAPC models are pivotal in the analysis of viral skin diseases as they enable precise forecasting of trends and risk assessment, thereby guiding targeted public health interventions and resource allocation (42). Our BAPC analysis predicts a decrease in ASIR, ASPR, and ASDR in the upcoming years. Nevertheless, the projections indicate that the overall burden of viral skin diseases is anticipated to rise across all metrics for both genders, with males consistently exhibiting higher rates than females. This trend highlights a growing public health concern, emphasizing the need for sustained efforts and resource allocation in this domain.

The escalating burden and prevalence of viral skin diseases can be ascribed to several factors. Firstly, the prolongation of lifespans has led to an increase in the susceptible population (43). Secondly, the enhanced exposure to adverse environmental conditions, including UV radiation and air pollution, has exacerbated the risk of developing skin diseases (44, 45). Additionally, the advancement in diagnostic techniques has resulted in better detection and reporting of skin diseases.

Gender differences also emerge as a significant factor. Males are more likely to engage in behaviors that heighten the risk of skin diseases, such as a higher prevalence of smoking (46), alcohol consumption (46), unprotected sex (47), and outdoor activities without adequate sun protection (48, 49). Additionally, due to their representation in industries like construction and agriculture, males are more frequently exposed to occupational hazards like carcinogens, chemicals, and other skin irritants (50, 51).

These insights underscore the urgency of raising awareness about the varied burden of viral skin diseases and promoting the development of targeted prevention and treatment strategies. Allocating appropriate health resources to address this growing concern is paramount (31).

While this article presents valuable insights into the global burden of viral skin diseases, it is essential to note some limitations. Firstly, the absence of subtype-specific data hampers a detailed analysis of the burden associated with different types of viral skin diseases, which could provide policymakers with more granular information. Secondly, the data quality is affected by issues such as underreporting, heterogeneity in data sources, and discrepancies in the definition of viral skin diseases across regions. Thirdly, while DALYs offer a valuable metric for assessing disease burden, they have limitations in capturing the full spectrum of impacts on individuals, families, and society (43). Nonetheless, as the GBD research framework continues to evolve, these limitations are anticipated to be addressed over time.

## 5 Conclusion

In summary, our study expands on the research conducted by Himed et al., who examined the worldwide burden of viral skin diseases until 2019 (36), with a primary focus on prevalence and YLDs concerning GDP. By providing updated and detailed data, our study offers vital insight into the global burden of viral skin diseases. These findings emphasize the need for ongoing surveillance and customized intervention strategies to effectively manage and lessen the burden of viral skin diseases globally. The updated information presented here reinforces the critical role of targeted public health measures in addressing these pervasive conditions and mitigating their impact on health systems worldwide.

## Data Availability

Publicly available datasets were analyzed in this study. This data can be found at: https://vizhub.healthdata.org/gbd-results/.

## Glossary

GBD: Global Burden of Diseases
DALYs: Disability-Adjusted Life Years
ASR: Age-Standardized Rate
ASIR: Age-Standardized Incidence Rate. This is the number of new cases of a disease in a standardized population, adjusted for age differences. It allows for comparisons of how often a disease occurs between populations with different age structures, usually expressed per 100,000 people
ASPR: Age-Standardized Prevalence Rate. This shows the proportion of people affected by a disease at a specific point in time, adjusted for age. It includes both new and existing cases, and is also measured per 100,000 people, which helps compare how widespread a disease is across populations of varying age structures
ASDR: Age-Standardized DALY Rate. This represents the rate of Disability-Adjusted Life Years (DALYs), which combines the years lost due to early death and the years lived with disability from a disease. It is age-adjusted and expressed per 100,000 people, giving an estimate of the overall burden of the disease in different populations
SDI: Socio-Demographic Index
APC: Age-Period-Cohort
BAPC: Bayesian Age-Period-Cohort
YLLs: Years of Life Lost
YLDs: Years Lived with Disability
GHDx: Global Health Data Exchange
ICD-10: International Classification of Diseases, 10th Revision
ST-GPR: Spatio-Temporal Gaussian Process Regression
EAPC: Estimated Annual Percentage Change
MCMC: Markov Chain Monte Carlo
INLA: Integrated Nested Laplace Approximation
CI: Confidence Interval
IMF: International Monetary Fund
UNESCO: United Nations Educational, Scientific and Cultural Organization
UI: Uncertainty Interval
