# Supplementary figures and images for: Global burden of viral skin diseases from 1990 to 2021: a systematic analysis for the global burden of disease study 2021
Source: Front Public Health. 2025 Feb 19;13:1464372. doi: 10.3389/fpubh.2025.1464372 (PMC11879981; doi:10.3389/fpubh.2025.1464372)

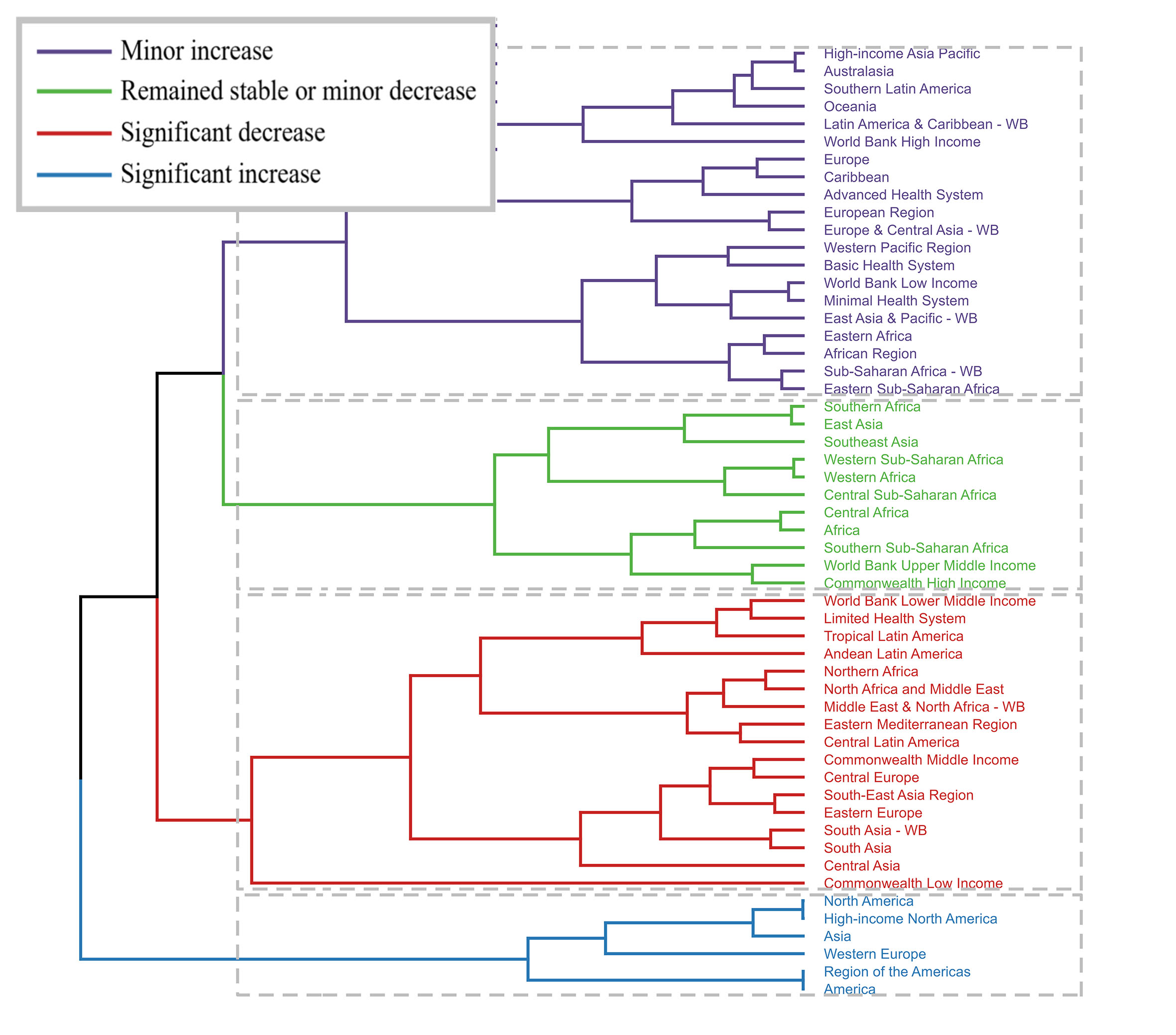

Supplement: Supplementary file 1 [file Data_Sheet_1.zip › 1464372_SupMaterial/90-21GBD region.JPEG]
